# Prediction of Microstructure for AISI316L Steel from Numerical Simulation of Laser Powder Bed Fusion

**DOI:** 10.1007/s12540-022-01168-x

**Published:** 2022-05-12

**Authors:** Maria Beatrice Abrami, Marialaura Tocci, Muhannad Ahmed Obeidi, Dermot Brabazon, Annalisa Pola

**Affiliations:** 1 Dipartimento di Ingegneria Meccanica e Industriale, Università Degli Studi di Brescia, Via Branze 38, 25123 Brescia, Italia; 2 I-Form, Advanced Manufacturing Research Centre, School of Mechanical and Manufacturing Engineering, Dublin City University, Glasnevin, Ireland

**Keywords:** Microstructure prediction, Cellular microstructure, Microhardness prediction, Numerical simulation, 316L stainless steel, Laser powder bed fusion

## Abstract

**Abstract:**

Laser powder bed fusion (L-PBF) success in the industrial scenario strongly depends on the ability to manufacture components without defects and with high building rates, but also on the ability to effectively control the microstructure to gain the required properties in the final component. In this regard, the recently developed numerical simulation software of L-PBF technologies can represent an effective tool, since many of them provide solidification data (i.e. temperature gradient and cooling rate) useful for microstructure prediction. In this work, a numerical model was applied to simulate the processing of four single scan tracks of 316L stainless steel processed with different parameters. Temperature and cooling rate around the melt pool were extracted from the numerical model and used to estimate the microstructure cellular arm spacing and the microhardness. Experimental measurements were then compared with the estimated values revealing good agreement. The good agreement between experimental and estimated values shows the advantages of the proposed method for microstructure and microhardness prediction based on numerical modelling as a useful resource for process optimization according to the required final microstructural features.

**Graphical Abstract:**

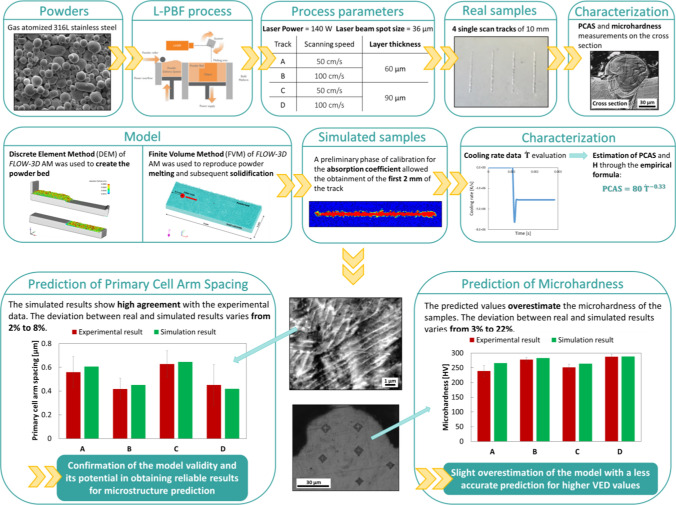

## Introduction

Laser Powder Bed Fusion (L-PBF) is the most important additive manufacturing (AM) technology for metals. During this process, the melting of a powder layer occurs due to the localized action of a laser moving according to predefined paths. L-PBF is characterized by cooling rates of about 10^6^ K/s, significantly higher than those of casting processes that are in the range of 10–10^2^ K/s [1] and those of laser cladding deposition (LCD) of about 10^2^ K/s [2]. Also, the thermal gradient involved in this process is extremely high, due to the localized action of the laser [3]. In this regard, it is well known that the cooling rate and the thermal gradient are the key parameters controlling the final microstructure [4, 5]. Therefore, the L-PBF rapid solidification causes significant changes in alloy microstructure as compared to conventional processes, e.g. the onset of structural constituents, the generation of highly refined microstructure, the suppression of solid-state diffusion or the production of non-equilibrium or meta-stable phases, resulting in different mechanical properties [6].

Numerous researches have been carried out using numerical analysis aimed at identifying not only optimal process parameters but also at predicting the final microstructure starting from solidification data. This is important because microstructural features are responsible for the mechanical properties of the material and therefore can be used to estimate the performance of the component in service. In detail, a correlation between process parameters and localized thermal characteristics (temperature and cooling rate) and solidification parameters (thermal gradient G and solidification rate R) was done by Huang et al. [7] in order to get an effective microstructure prediction in the directed energy deposition (DED) process. Solidification maps were used for microstructural prediction for both 316L steel and Inconel 625 alloy and results were compared with experimental observation, showing good agreement. Shahabad et al. [8] calibrated a heat source model based on experimental measurements of melt pool width and depth for Hastelloy X single track in L-PBF process. Thermal behavior was then analyzed from the numerical model in order to provide a microstructure prediction and to analyse its variation in relation to the different process parameters. Finally, microstructural features calculated from software output were compared to the experimentally measured ones, revealing good agreement and consequently the reliability of the model.

Several of these studies are based on the austenitic stainless steel 316L, which commonly exhibits a cellular microstructure instead of a dendritic one when produced via L-PBF, due to the high temperature gradients and cooling rates during solidification [2, 4, 5]. In particular, primary cellular arm spacing (PCAS) is the characteristic feature of this microstructure and it is the key parameter controlling strength, according to the Hall–Petch relationship. PCAS is defined as the average distance between centerlines of adjacent primary cells [9]. Moreover, cellular microstructure seems to be controllable by changing the laser power [5]. This fact has been confirmed by a study of Vecchiato et al. [10], which pointed out a coarsening of the cellular microstructure as the energy absorbed increases, directly affected by laser power. A thermal model was developed by Scipioni Bertoli et al. [5] in order to express solidification parameters G and R as a function of the process parameters ratio P/v (laser power/scan speed). This provides a guide for the selection of the process parameters for 316L steel manufactured via L-PBF. They detected that G/R ratio, related to the microstructure morphology, remains in the range of 100–200 K s/mm^2^ for most of P and v combinations used in the L-PBF technique, demonstrating the stability of the columnar cellular microstructure in the process. A previous study on L-PBF manufactured 316L steel [11] compared the experimentally determined cooling rates with values provided by numerical simulations and revealed good agreement. Moreover, they observed accordance between the predicted cell spacing (based on cooling rates from numerical model) and previous data in literature. Tang et al. [12] analyzed the humping defects in 316L single scan track with a mathematical model and revealed the arise of irregular humps at high scanning velocities, which was validated against experiments. Furthermore, they studied the flow kinetics to understand the physical origins of humping formation.

Besides laser power and scanning speed, also layer thickness constitutes a fundamental process parameter, as the layer is the primary element on which the deposition is based. In fact, a correct layer thickness determination is the key factor to achieve penetration of the fused area in the substrate, assuring good bonding between the subsequent layers and the absence of lack of fusion defects. Moreover, it was experimentally documented that layer thickness directly affects the build rate: high layer thickness values are desirable to reduce the build time, but they involve melt pool instability which in turn produces non-repeatable track geometry [13–15]. Additionally, Cao [16] examined the effect of layer thickness on porosities in L-PBF process of 316L using numerical simulation, and highlighted that a large layer thickness results in porosities in the bottom of the track due to insufficient melting, while a small layer thickness implies low process efficiency. However, there are no numerical modelling studies that consider the effect of layer thickness on the cooling rate of the track and the resulting microstructure. In this context, the purpose of this work consists in the study of the influence of layer thickness on the microstructure of AISI 316L stainless steel using thermal and solidification data (i.e. temperature and cooling rate) obtained by numerical simulation. Furthermore, the effect of other process parameters on temperature distribution and microstructure were extensively evaluated. Microhardness was also predicted with consideration of the cooling rate obtained for the different investigated conditions. There is a previous work reporting on microhardness evaluation of 316L single scan tracks but not bulk samples [17], however it is not focused on the microhardness prediction using numerical modeling. In this regard, the present study proposes a new method to improve L-PBF efficiency and to decrease the production costs by using simulations. The optimization of process parameters themselves is performed numerically, reducing the experimental tests required to attain the optimal produced sample properties. The commercial software *FLOW-3D AM* was used and results were then validated with experimental microstructural characterization and microhardness measurements of AISI 316L single scan tracks produced by laser powder bed fusion.

## Experimental Procedure

### Samples Production and Characterization

**Fig. 1:**
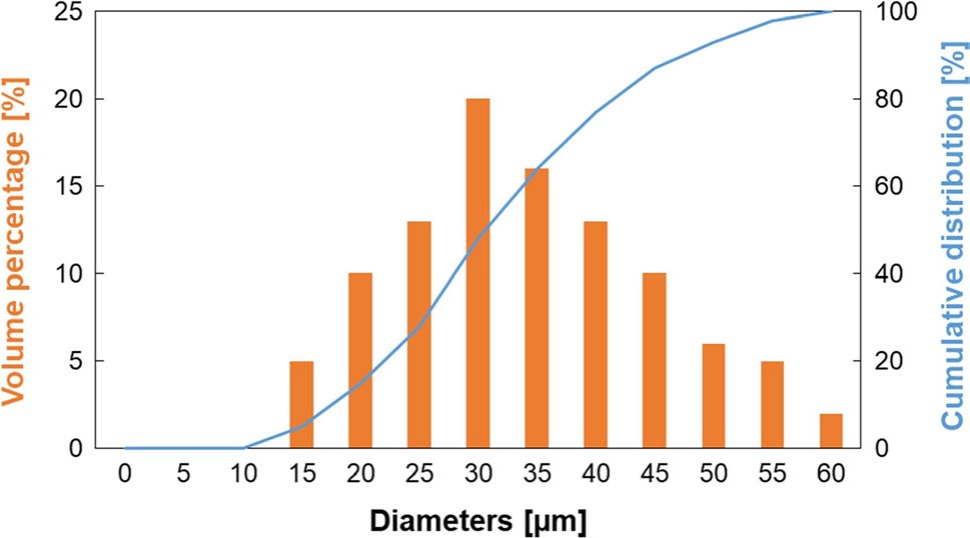
Particle size distribution of powders used

In this study, gas atomized 316L stainless steel powder supplied by Carpenter Additive was used to produce the samples. The chemical composition is shown in Table 1. The particle size distribution is reported in [18], it follows a Gaussian distribution, ranging from 15 to 60 µm, with D_50_ of 30 µm and D_90_ of 47 µm. PSD used in simulations was extracted from [18] and approximated with discrete intervals, as reported in Fig. 1.

**Table 1:** Mean chemical composition of the 316L stainless steel powders (wt%)

C	Cr	Cu	Mn	Mo	Ni	N	O	P	Si	S	Fe
0.02	17.6	0.02	0.66	2.38	12.5	0.09	0.03	0.007	0.65	0.006	Bal.

Single scan tracks of 316L steel were produced using an Aconity MINI build chamber, a powder bed fusion system equipped with a 200 W fiber laser of 1068 nm wavelength. The maximum build volume is 140 mm diameter and 200 mm height.

Different process parameters were used for the single-track manufacturing, as reported in Table 2. A constant value of 140 W was chosen for the laser power, while scanning speed was varied using two values, i.e. 50 and 100 cm/s. The same combinations of laser power and speed were then applied at two layers with different thicknesses (60 and 90 µm) in order to evaluate the effect of the layer thickness on the solidified tracks, as the main purpose of the work. Each scan was 10 mm in length, while the laser spot diameter was of 36 µm.

**Table 2:** Different process parameters combinations used

Track name	Laser power (W)	Scanning speed (cm/s)	Layer thickness (µm)
A	140	50	60
B	140	100	60
C	140	50	90
D	140	100	90

Top surface morphology was examined using a LEICA DMS300 digital microscope. In order to observe the cross section of the tracks, samples were mounted in acrylic resin and grinded up to 1 mm from the beginning of each laser track. The cross section thus obtained was polished up to mirror finishing.

The observation of the cross sections at high magnification was performed with a scanning electron microscope (SEM) Leo Evo 40XVP. Samples were analyzed after chemical etching with aqua regia reagent (3:1 volume mixture of HCl to HNO_3_) for 1 min at 50 °C to identify the cellular structure. Measurements of PCAS were carried out on SEM images. The experimental PCAS average was calculated as the average of three values measured in different grains near the center of the melt pools.

Finally, Vickers microhardness measurements were performed with a Mitutoyo HM-200 hardness tester with an applied load of 10 g for 20 s. Measurements were carried out in the melt pool area, in particular three values were obtained and their average together with standard deviation were calculated. Indentations were made at a distance from each other and from the edge of the track equal to 2.5 times the measurement of the average diagonal of the indentation.

### Numerical Model

In the present study, the commercial software *FLOW-3D AM* was used to simulate L-PBF manufacturing of 316L stainless steel single scan tracks and to predict solidification data. *FLOW-3D AM* consists in two modules. The creation of the powder bed was modelled with DEM (Discrete Element Method), while powder melting and subsequent solidification were simulated with the WELD module (Finite Volume Method, FVM). Details on the models used and equations specifically solved by *FLOW-3D AM* software can be found in [19].

**Fig. 2:**
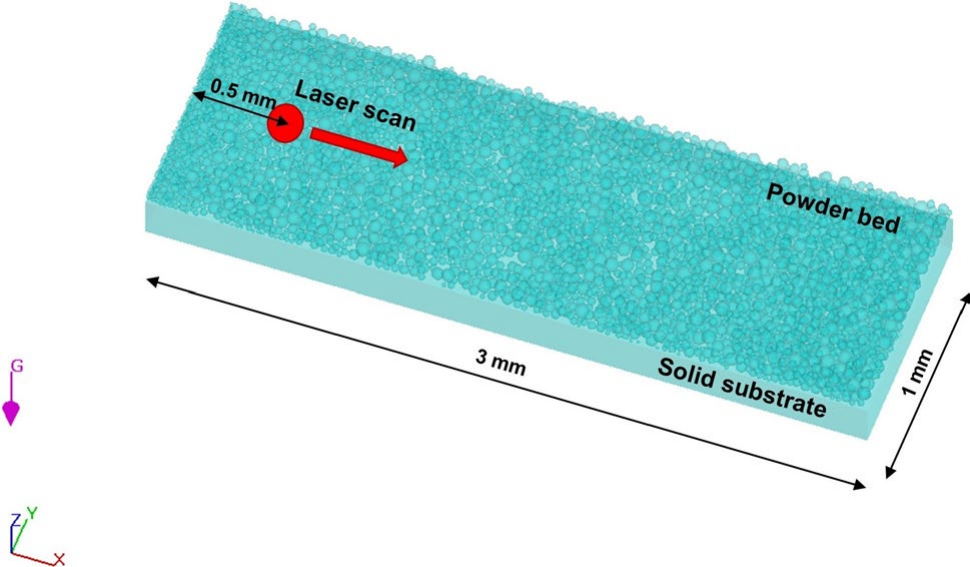
Configuration of the thermo-fluid model

In order to simulate the laser scanning phase, a domain of 3 × 1 × 0.4 mm ($x$ × $y$ × $z$) was created, as displayed in Fig. 2, consisting of both the powder bed and the substrate. The substrate is of the same material of the powder layer. Laser travels along $x$ direction and a scan length of 2 mm was simulated for each combination of process parameter (Table 1). A fine mesh with cell size of 6 µm is applied to the central area where there is the scan track to better monitor the shape and size of the melt pool, while a coarser one with cell size of 15 µm is used for the area where no melting takes place. In addition, a mesh of 40 µm is used in the surrounding area for thermal diffusion calculations. The total number of cells is about 1.2 million. The choices of the domain size and mesh dimensions are the result of optimization steps to achieve reasonable simulations results in terms of time and precision, together with a correct evaluation of heat exchange phenomena.

Temperature-dependent thermal properties were taken from the literature [20–22]. Other properties of 316L stainless steel are reported in Table 3 [20].

**Table 3:** Material properties for stainless steel 316L [20]

Property	Value
Liquidus temperature (K)	1697.15
Solidus temperature (K)	1674.15
Boiling temperature (K)	3090
Latent heat of fusion (J/kg)	2.6 × 10^5^

It is well known that the melt pool morphology and the solidified track are strongly affected by the Marangoni effect. Therefore, in the present model this was taken into account through the following equation, which describes the change of surface tension as a function of temperature:

$$\sigma \left( T \right) = \sigma_{0} + \frac{d\sigma }{{dT}}\left( {T - T_{L} } \right)$$

where $\sigma_{0}$ is the surface tension at liquidus (melting) temperature ($T_{L}$) and $d\sigma /dT$ is the surface tension gradient. The coefficients used in this equation can be found in [22].

The laser input is modelled as a moving heat flux with Gaussian distribution:

$$Q = \frac{{A_{b} P_{L} }}{{\pi R_{s} }}{\text{exp}}\left( { - \frac{{\left( {x - x_{s} } \right)^{2} + \left( {y - y_{s} } \right)^{2} }}{{R_{s} }}} \right)$$

where $Q$ is the instant surface heat flux, $A_{b}$ is the absorption coefficient, $P_{L}$ is the laser power, $R_{s}$ is the laser radius, and $x_{s}$ and $y_{s}$ are the coordinates of the laser beam center.

A preliminary phase of calibration for the absorption coefficient was performed by comparing simulated and real track top view morphology in order to achieve reliable results. The calibration procedure in more detail can be found in [23]. The absorption coefficient is needed for simulation setting, and quantifies the energy absorbed by the substrate. The absorption coefficient was varied four times using the following values: 0.3, 0.4, 0.5, 0.6. The best value turned out to be 0.6, as also reflected in literature [24]. To strengthen this assumption, melt pool width and depth values obtained from the numerical model were compared with those experimentally measured. In detail, simulation measurements were carried out in 8 different sections, while experimental measurements in at least 3 different sections after chemical etching. Then, the average value and standard deviation were calculated for each track and compared to each other.

Regarding the purpose of the work, temperature and cooling rate distributions were extracted both as a function of time and space along $z$ direction in order to study the effect of scanning speed and layer thickness during the process. From the cooling rate value obtained from simulations, PCAS was calculated as follows:

1 $$\lambda_{1} = 80\dot{T}^{ - 0.33}$$

where $\lambda_{1}$ is PCAS and $\dot{T}$ is cooling rate of the interface of solid/liquid during solidification [2, 8]. This empirical equation has been frequently used in previous studies for explaining the relationship between PCAS and cooling rate for austenitic stainless steels [2]. In this work, Eq. (1) was used to predict the microstructural features from thermal data from numerical modelling. For validation, PCAS predicted values were compared with the average value of those measured experimentally for each sample.

The grain width *d* was also calculated according to following relationship [2]:

2 $$d = \frac{{5.9 \times 10^{9} }}{{\left( {\sqrt {\dot{T}} } \right)^{3} }} - \frac{{2.6 \times 10^{7} }}{{\left( {\sqrt {\dot{T}} } \right)^{2} }} + \frac{{4.2 \times 10^{4} }}{{\sqrt {\dot{T}} }} + 1.6$$

Subsequently, starting from grain width *d* it was possible to predict the microhardness *H* for each sample [2]:

3 $$H = 152 + 498d^{ - 0.5}$$

This relationship was obtained experimentally for 316L processed via L-PBF, and it confirms the linear dependence between grain-size and microhardness expressed in the Hall–Petch equations. The predicted microhardness values were then compared with the average value of those measured experimentally for each sample.

## Results and Discussion

### Experimental Results

#### Track Morphology

Single scan tracks were analyzed by digital microscopy to observe the main morphological features of the top view. As reported in the literature [25], tracks can be classified depending on their shape in three main categories: continuous, irregular, or balling. Continuous tracks appear smooth and almost constant, irregular shape is characterized by a non-homogeneous and uneven appearance and balling morphology exhibits a series of barely connected metal beads.

**Fig. 3:**
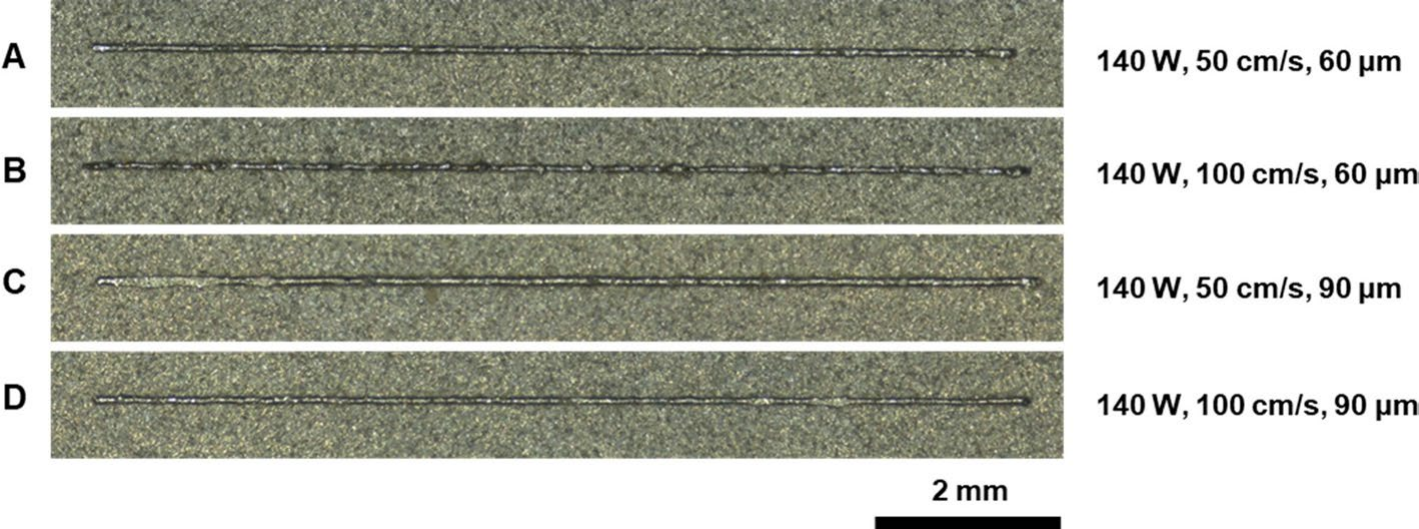
Top view morphology of the single tracks with the corresponding process parameters

According to this classification, from the observation of Fig. 3, it appears that all the tracks exhibit a continuous and stable shape along the entire 10 mm length. The only track showing small deviations from regularity is track B; nevertheless, these are negligible compared to the global track trend.

A decrease in track width can be observed as scanning speed increases, as in these cases the energy carried to the powder bed decreases because of the lower laser-metal interaction time.

Nor irregular or balling morphologies are observed, revealing that the energy carried to the powder bed is sufficient to obtain a regular powder melting [25]. The continuous regime is the one desired in the manufacture of L-PBF parts, since it allows good bonding between adjacent tracks, and results in minimal level of porosity.

#### Microstructural Morphology

**Fig. 4:**
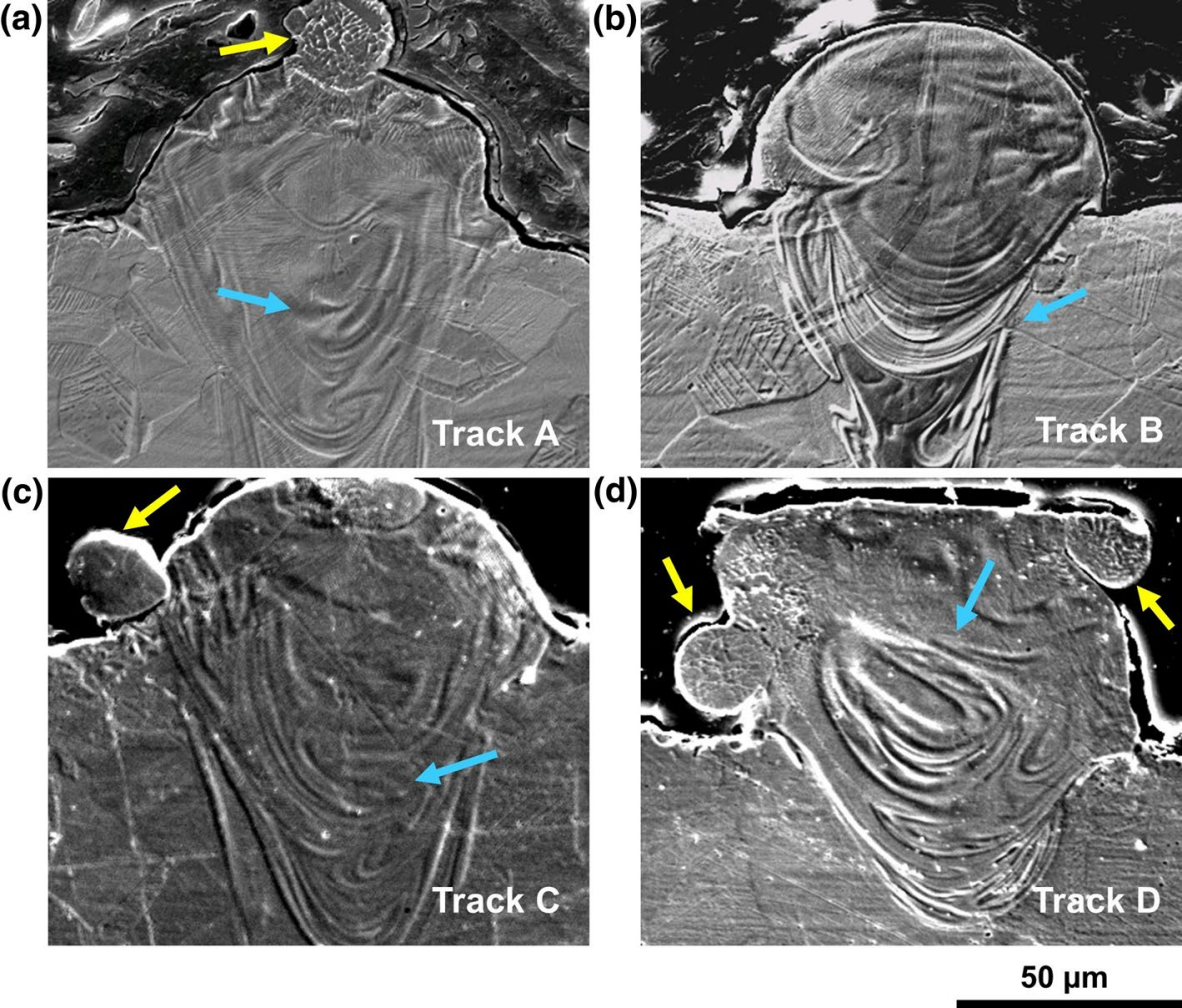
SEM images revealing melt pool presence for: **a** track A, **b** track B, **c** track C and **d** track D. The semi-molten powders are highlighted by the yellow arrows (lighter), while the turbulent flow appearance is pointed out by the blue arrows (darker)

The cross sections of the scan tracks were observed using SEM microscope. Images are reported in Fig. 4 and reveal the presence of the melt pool and solidification microstructure. The upper end of melt pools of samples A, C and D are characterized by sintered or semi-molten powders (highlighted by yellow arrows), probably due to spatter phenomena or insufficient energy carried to the powder bed. Furthermore, in all samples it is possible to detect phenomena related to the reverse Marangoni flow (highlighted by blue arrows). This consists in a centripetal flow moving the molten metal from the outside to the center of the melt pool and results in a turbulent flow appearance once solidified. This phenomenon was previously detected in another study regarding laser powder bed fusion process for a CoCr alloy [26].

**Fig. 5:**
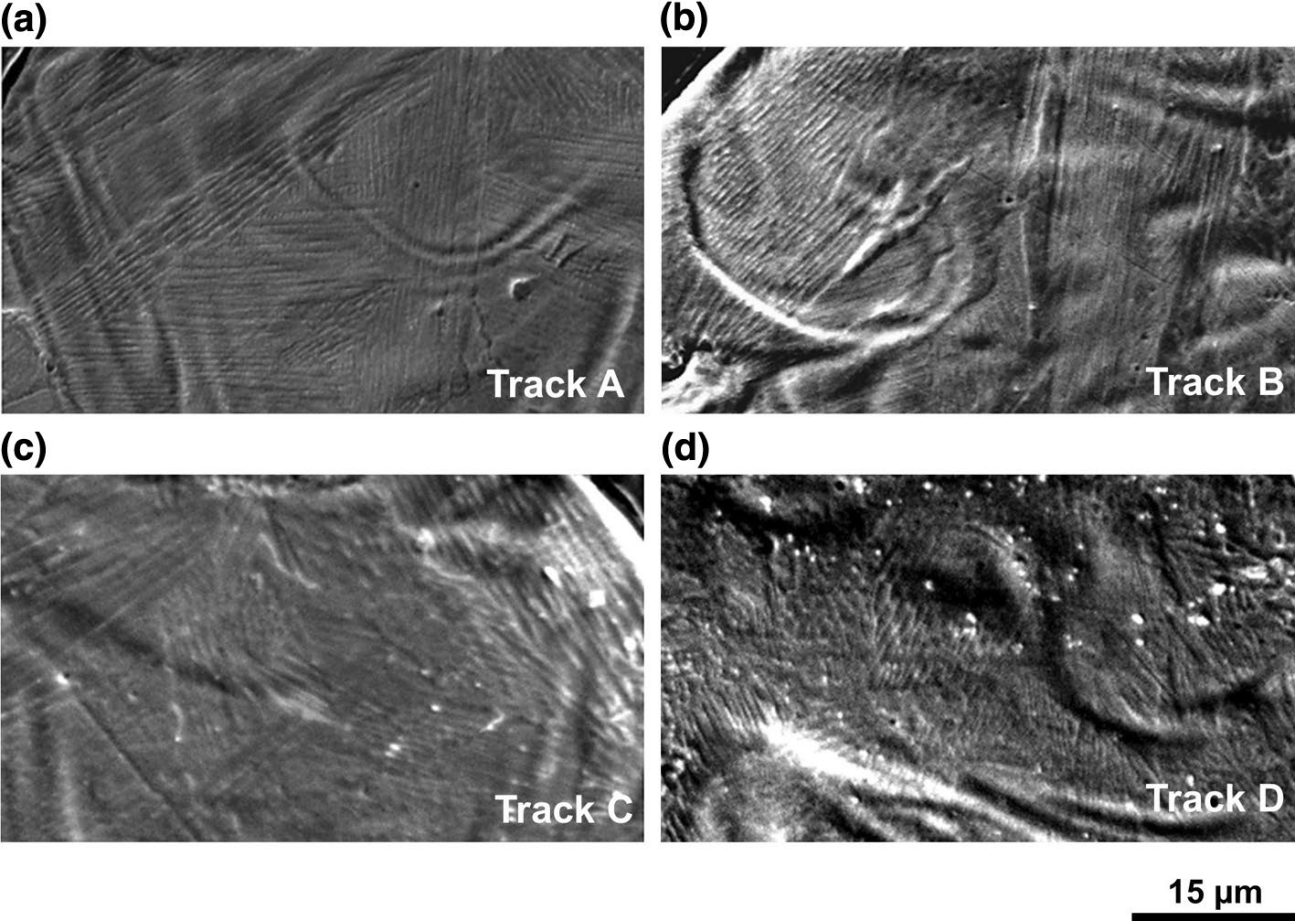
SEM images showing the cellular structure inside the melt pool of: **a** track A, **b** track B, **c** track C and **d** track D

As can be better detected in the melt pool magnifications of Fig. 5, all the samples are characterized by a cellular microstructure. This is the result of the high cooling rates during solidification in L-PBF process. In fact, it is known that the high cooling rates of this process (about 10^6^ K/s [1]) lead to a microstructural refinement compared to traditional processes. Moreover, the high temperature gradients can lead to a strong Marangoni convention [27, 28], which explains the turbulent morphology previously mentioned. Furthermore, it can be noted that the submicrometric cells are contained in grains elongated in the direction of the maximum heat flux, perpendicular to the melt pool boundary.

PCAS average values are reported in Table 4, together with the process parameter of each track. Despite the significant standard deviations, scanning speed appears as the main parameter affecting solidification conditions and PCAS. A decrease in PCAS is detected as scanning speed increases, for both the layer thicknesses considered. The effect of the layer thickness is instead negligible as PCAS values appears almost constant when thickness changes.

**Table 4:** Corresponding process parameters and PCAS for each track

Track name	Laser power (W)	Scanning speed (cm/s)	Layer thickness (µm)	PCAS (nm)
A	140	50	60	558 ± 130
B	140	100	60	416 ± 91
C	140	50	90	627 ± 112
D	140	100	90	451 ± 171

### Simulation Results

#### Track Morphology and Melt Pool Size Comparison

**Fig. 6:**
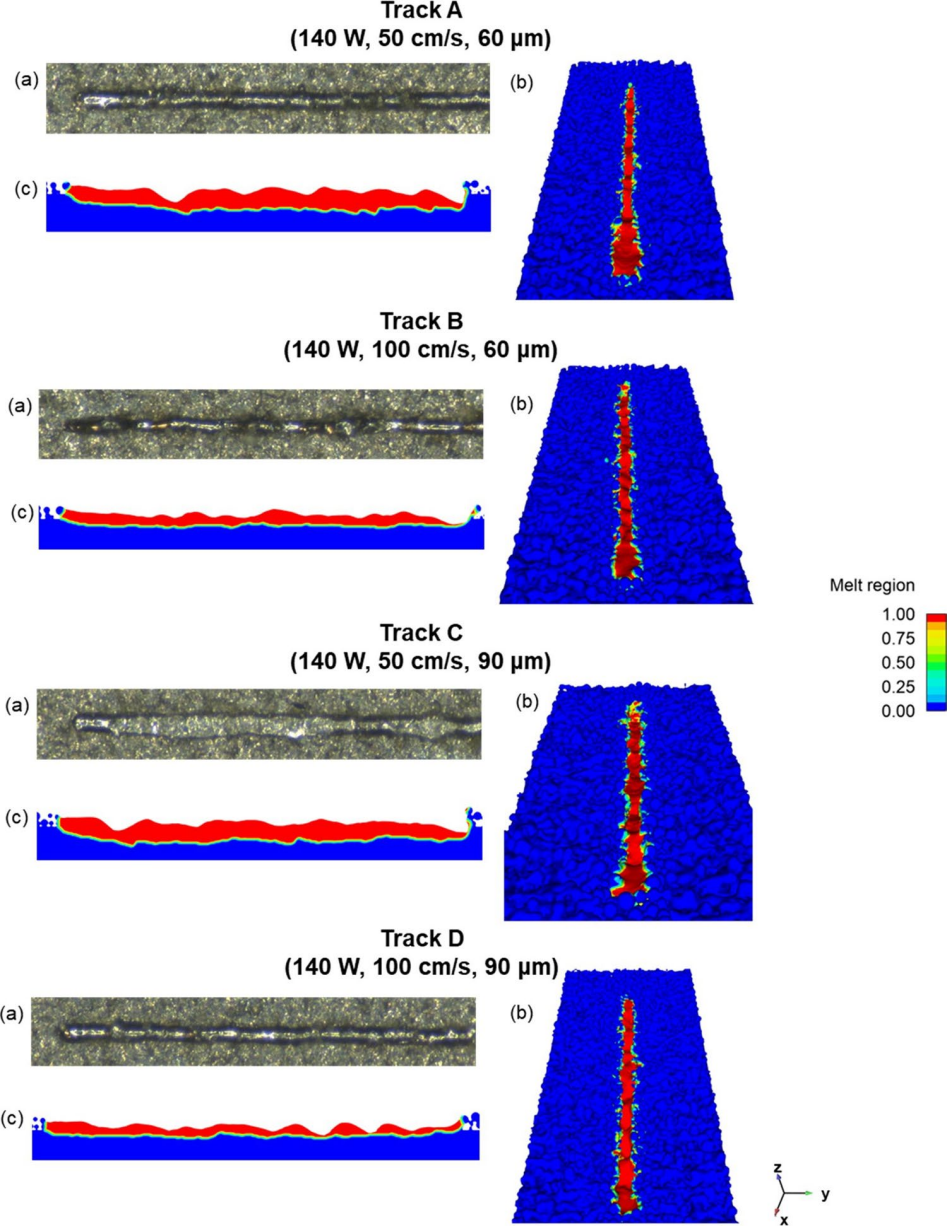
Comparison between experimental and simulation results, for each processing parameters: **a** experimental result, **b** simulated 3-dimensional view, **c** sectional view (longitudinal)

A global comparison between the first 2 mm of simulated and real tracks is shown in Fig. 6. The comparison between the top view of the track is shown in Fig. 6a, b, where good agreement between results can be detected, as the morphology of the real track (a) is well predicted by simulations (b). This fact denotes a correct calibration of the simulation parameters, included the absorption rate, set to 0.6 for these combinations of process parameters, as above mentioned.

Furthermore, the prediction of the central longitudinal sections for each sample are reported in Fig. 6c. An acceptable penetration in the substrate can be detected for all the combinations of process parameters. For a scanning speed of 50 cm/s (track C and D), penetration depth appears more irregular and deeper. In some points, in fact, depth seems to reach values that could lead to a keyhole melting mode. Keyhole melting mode occurs when evaporation temperature in the melt pool is exceeded and metal vaporization can take place. In this case, a deep melt pool is created as a result of the recoil pressure generated in the molten metal. The melt pool shape is that of a keyhole, with a high depth/width ratio. Keyhole melting mode leads to a major presence of defects as spatter or keyhole porosities, and for this reason it is not the desirable melting mode. Conduction is the other melting mode, and it takes place when the peak temperature is lower that the boiling one. It is characterized by melt pool with roughly semi-cylindrical shape and greater stability of the track, which makes it the preferable melting mode [25].

On the other hand, scanning speed of 100 cm/s is associated with a shallow penetration in the substrate, which denotes a more stable conduction melting mode. This is verified for both 60 and 90 μm of layer thickness, pointing out a major effect on penetration depth of scanning velocity than that of layer thickness.

In addition, simulation and experimental values of melt pool width and depth were compared. Results of the comparisons are reported in Table 5, where the predicted melt pool dimensions show a reasonable agreement with those experimentally measured. In detail, the deviation between the simulated and experimental values ranges from 7 to 22% for width, while from 10 to 16% for depth. The slight discrepancy between simulated and experimental values (as for example for the width of sample B) can be linked to the fact that the melt pool morphology is strongly variable within the track length, as can be seen in Fig. 6. Therefore, it is possible to state that these comparisons demonstrate the reliability of the numerical simulation used in this study.

**Table 5:** Width and depth comparisons between simulation and experimental results

	A		B		C		D	
	Width	Depth	Width	Depth	Width	Depth	Width	Depth
Simulated measurements (µm)	98 ± 7	79 ± 9	97 ± 4	35 ± 7	109 ± 15	71 ± 10	101 ± 9	33 ± 6
Experimental measurements (µm)	92 ± 3	88 ± 14	80 ± 4	31 ± 8	107 ± 4	85 ± 4	91 ± 7	38 ± 4

By comparing the experimental values, a strong decrease in depth as laser scanning speed increases can be observed. This can be attributed to the lower energy provided to the powder bed. In addition, no effect is noted as layer thickness increases, denoting a minor influence of this parameter rather than scanning speed on melt pool depth. On the other side, only a slight decrease in width can be detected as the scanning speed increases, while a slight increase in width can be observed as the layer thickness increases.

#### Temperature Distribution

**Fig. 7:**
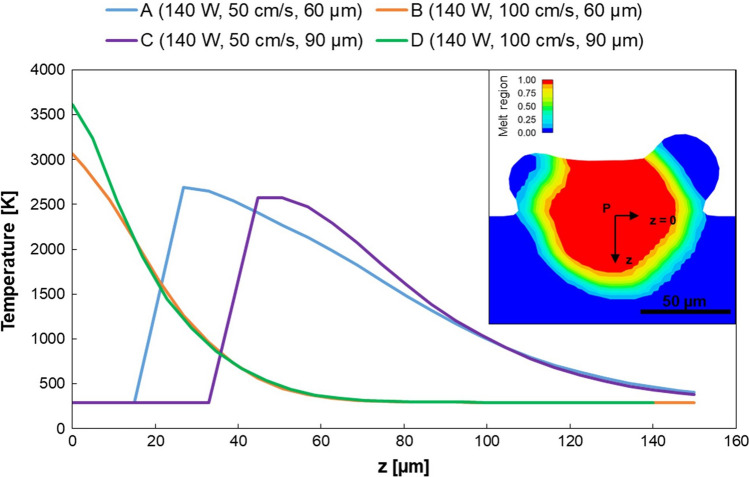
Temperature distribution within the melt pool and a representative section showing the location where the displayed temperature-displacement line probe profiles were taken (from point P)

**Fig. 8:**
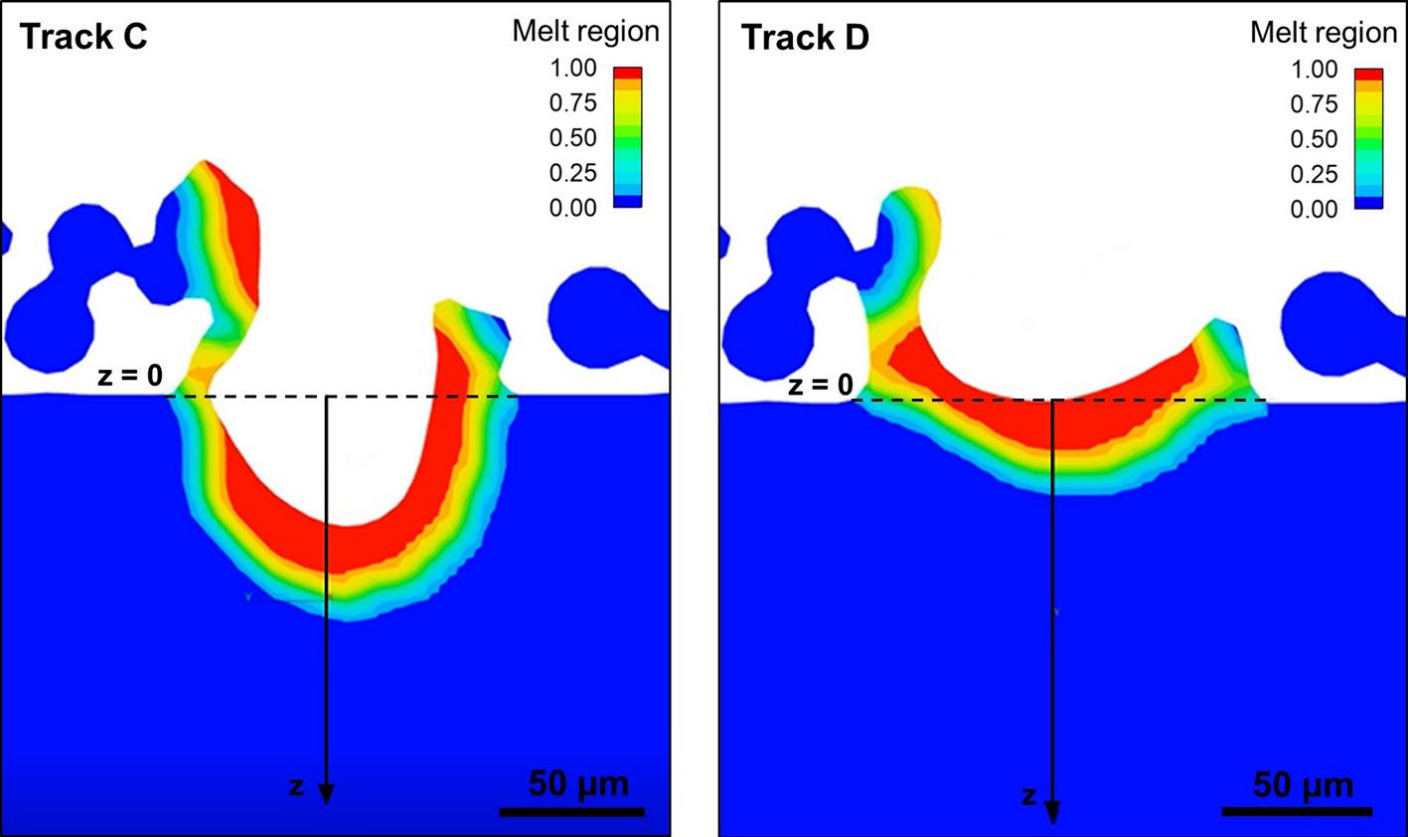
Example of depression zone of track C, manufactured with 50 cm/s of scanning speed and the corresponding track at 100 cm/s (track D)

Temperature distributions for selected cross sections were extracted as a function of the distance from the substrate $z$ in the instants immediately after the laser scanning. Results are reported in Fig. 7 together with a section of sample B taken as representative image to show the location where the temperature-displacement profiles were recorded. For both the tracks manufactured with 50 cm/s (A and C), a depression zone can be detected in the first part of the substrate, also a few moments after the laser scanning. Here in fact, the temperature calculated by the model is the room temperature, which means that there is no material in these areas. An example of depression zone identified is reported in Fig. 8, together with the corresponding track at higher scanning speed, which reveals a less deep melt pool with no depression areas.

As Khairallah et al. [22] reported, depression zones are generally located at the laser spot as the result of the strong dynamical melt flow generated. In these instants, the dominant effect is the recoil pressure, which applies an increasing force normal to the melt surface accelerating the liquid away from the center. Therefore, the digging of the recoil pressure generates these considerable depressions as result.

Recoil pressure is in turn highly influenced by temperature [22]. This fact is confirmed in the present study, where depression zones persist after laser passage only for samples manufactured with lower scanning speed, where higher temperature are reached at the same substrate distance *z* (Fig. 7).

The persistence of depression zone can cause instability and turbulences, as the solid–liquid front is more variable inside the substrate. Moreover, a possible mechanism for pore formation is related to a vortex generated at the rear of the depressions and to the depression wall collapse [22]. Therefore, deep depressions, which could be due to extra heat deposited, should be avoided to decrease the pore arising.

Depressions are also related to the keyhole melting mode, as its driving force is the recoil one [22]. This confirms what noticed from the penetration depth detected in the longitudinal section of samples A and C (Fig. 6c) and already discussed above.

Comparisons between track A and B and between track C and D can be useful to analyze the influence of the scanning speed. Neglecting the first part of the curves A and C, related to the depression formation already discussed, it emerges that the lowest scanning speed results in higher temperature and higher penetration depth, as expected. In fact, with the same laser power, scanning speed is the parameter responsible for the energy carried to the metal, as it directly affects the exposure time of the powder bed to the laser, which is higher at lower laser scanning speed. Moreover, the slope of curves B and D appears steeper than A and C. This indicates a faster decrease in temperature as the depth increases (corresponding to a *z* increase in Fig. 7), as a result of the higher scanning speed used for the manufacturing of these samples.

The effect of layer thickness can be analyzed by comparing respectively sample A with C and sample B with D. In these cases, differences between temperature data at the same *z* are minimal: only a slight increase in temperature can be detected by increasing layer thickness in the first part of the curves, which then stabilizes at comparable values. This result agrees with a previous study [29] about the effect of layer thickness on residual stresses. In fact, an increase in layer thickness results in an obstacle in heat conduction to the substrate since a greater volume of particles is placed above the substrate itself. However, the difference is very weak and further analyses on larger layer thickness should be taken.

**Fig. 9:**
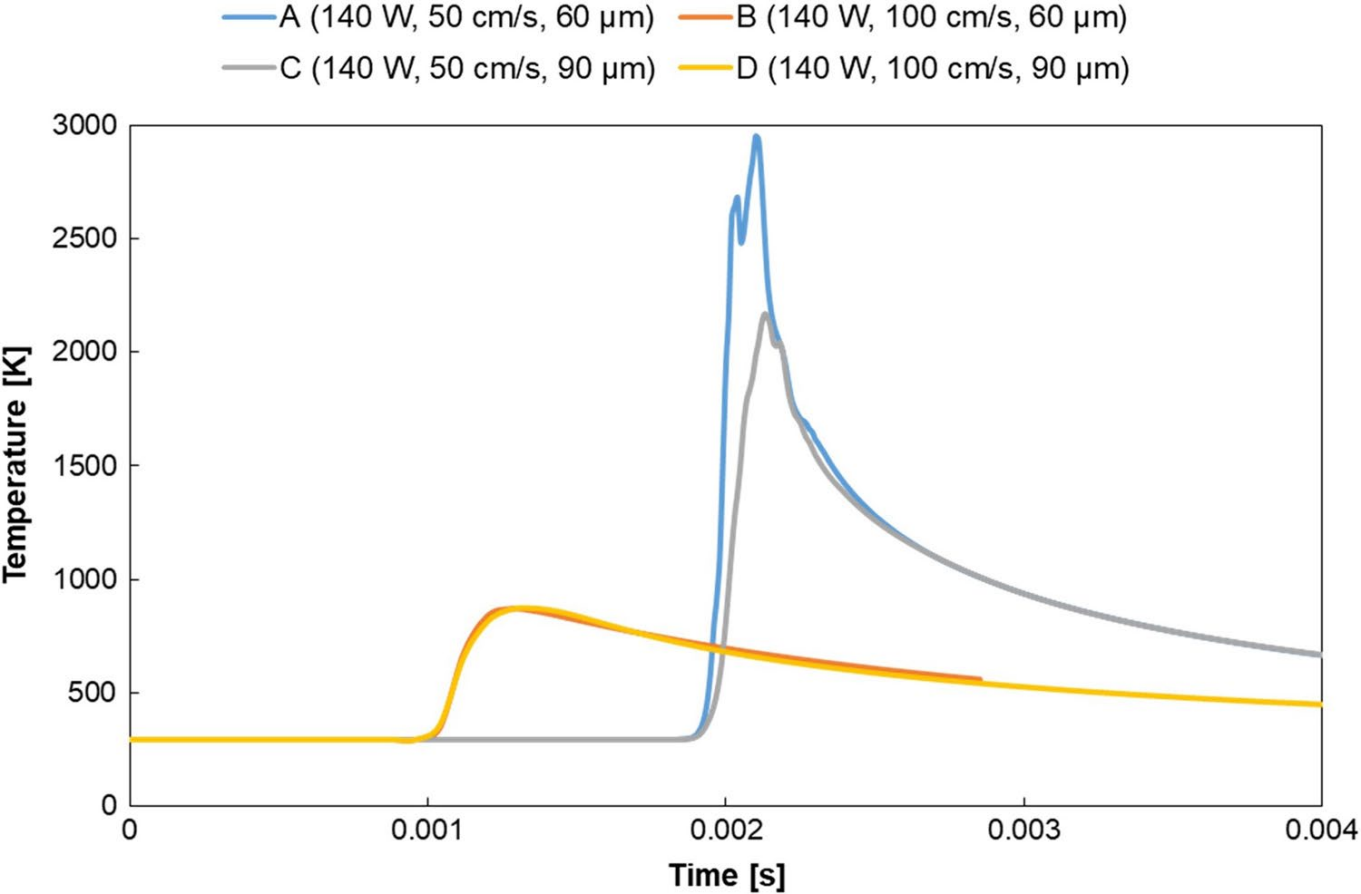
Temperature variation as function of time for a point located at z = − 60 μm from the substrate, for every track

To better understand the temperature experienced by the powder bed as the process parameters change, the temperature vs time plot is provided for a point 60 µm deep from the substrate to monitor the reheating of the underlying layer (Fig. 9). This allows observing changes in the same point by varying process conditions. As expected, melting occurs at the same time for samples with the same scanning speed (A and C, B and D). Moreover, the peak temperature reached is higher for samples manufactured with lower scanning speed (A and C), due to a greater amount of heat resulting from the higher interaction time between laser and powders. This can appear in contrast with Fig. 7, anyway it is necessary to remember that the temperature data in the first area of this plot are affected by the void created because of the depression zone, as previously widely discussed. However, the peak temperature values of tracks A and C reported in Fig. 9 cannot be considered as reliable. In these cases, in fact, in the moments when the laser passes over the point considered, the software calculation is directly affected by the depression zone presence. The depression zone exposes the point directly in contact with the laser, thus compromising the output extracted at 60 µm from the substrate and therefore the temperature values in those instants. Nevertheless, by comparing track A to C and track B to D, it is evident that the global trend is the same, meaning that there is no considerable temperature variation as layer thickness varies, for a point located deep into the substrate. This is also confirmed in the plot of Fig. 7, in which significant temperature variations are detected only near the surface.

#### PCAS and Cooling Rate Evaluation

**Fig. 10:**
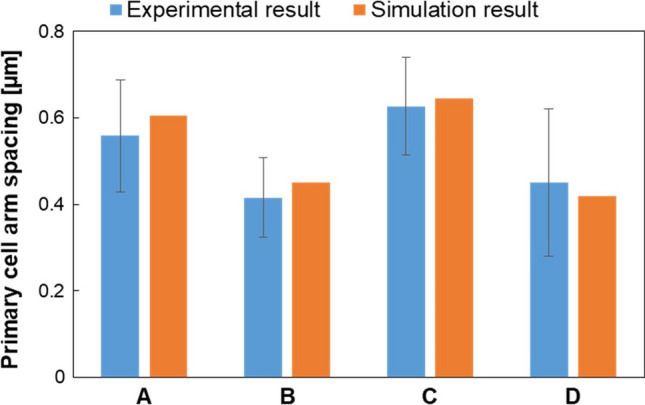
Comparison between experimental and predicted PCAS values for each sample

The PCAS was calculated using Eq. (1) based on cooling rate data extracted from the model. The obtained results were compared with the experimental average values of PCAS. Results of these comparisons are shown in Fig. 10. As can be seen, measured and calculated PCAS values are very close for each sample, in particular the deviation between the simulated and experimental values ranges from 2 to 8%. Moreover, it should be noted that the numerical result is always placed within the standard deviation of the experimental data. Therefore, an accurate agreement between simulation and experimental results can be detected for the different combinations of process parameters. This confirms both the good accuracy of Eq. (1) for the prediction of PCAS and the simulation reliability in predicting L-PBF cooling rate, allowing the numerical model validation.

**Fig. 11:**
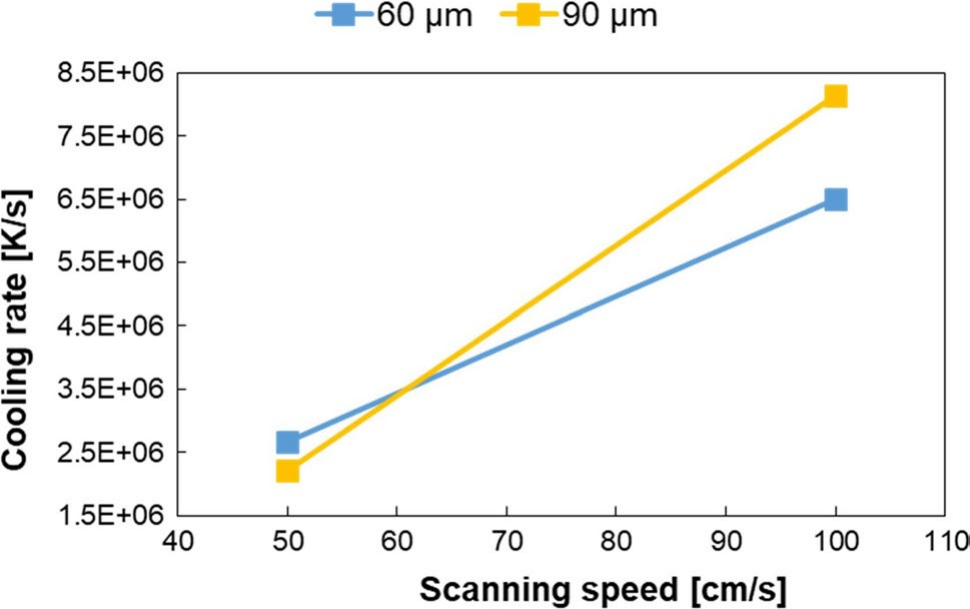
Cooling rate variation depending on scanning speed at different layer thicknesses

The cooling rate trend in relation to scanning speed, for both layers of 60 and 90 μm, is presented in Fig. 11. An increase in scanning speed results in a rise of cooling rate, for both the layer thicknesses. This is due to the lower energy carried to the powder bed generated by a reduction in laser-metal interaction time as the scanning speed increases. In this situation, the peak temperature reached by samples manufactured with higher scanning speed is lower (as reported in Fig. 9), consequently, since there is less heat to dissipate, these specimens will experience higher cooling rates.

Concerning the effect of layer thickness on cooling rate values, at low scanning speed it appears as minimal, while at higher scanning speed it is more pronounced. However, this difference results in a minimum variation of PCAS between samples B and D, as can be observed in Fig. 10, which implies that the influence of the considered values of layer thickness on microstructural features are negligible. Moreover, it is not possible to detect a uniform trend of cooling rate by changing the layer thickness.

As a result of these analyses, it emerges that scanning speed is the key parameter affecting cooling rate, if the laser power is constant.

#### Microhardness Evaluation

**Fig. 12:**
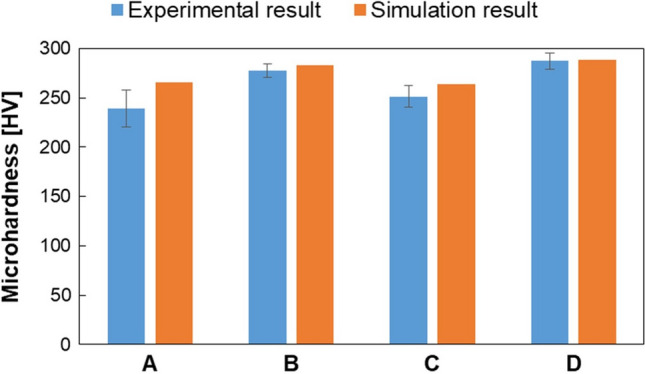
Comparison between experimental and predicted microhardness for each sample

Microhardness was measured inside the melt pool for each track. Then, the average values together with their standard deviations were calculated. In addition, microhardness was predicted by using Eq. (3), starting from grain width, calculated in turn from cooling rate data (Eq. 2). The obtained values were compared with the experimental average values of microhardness. Results are reported in Fig. 12.

As a general trend, the predicted values slightly overestimate the microhardness of the samples, and the deviation between real and simulated results varies from 1 to 11%. In particular, the deviation from the experimental results is the highest for sample A and C.

In this regard, it must be noted that the microhardness model proposed by Ma et al. [2] is based on samples manufactured with volumetric energy density values that range from 43 to 78 J/mm^3^, while in this work the volumetric energy density values used are reported in Table 6. It should be underlined that the volumetric energy density (VED) is given by the formula VED = P/dvt, where P is the laser power, d the laser spot diameter, v the scanning speed and t the layer thickness.

**Table 6:** Volumetric energy density values calculated for each track

Track	VED (J/mm^3^)
A	130
B	65
C	86
D	43

Samples A and C emerges as those manufactured with the highest VED and outside the range in which the model was previously verified by Ma et al. [2]. Therefore, it can be concluded that the predictive equation is sensitive to the values of volumetric energy density and it is less accurate with increasing energy density values. Further analyses on values higher than 78 J/mm^3^ should be performed to identify a more accurate and general model. This should be considered when using PCAS to predict material hardness.

In addition, it should be noted that the experimental microhardness values are in line to the PCAS values measured. In fact, sample B and D appear as those with higher microhardness, corresponding to the lower values of PCAS (Table 4). On the other side, sample A and C show lower microhardness values, due to a higher PCAS (Table 4), which indicates coarser microstructure. Also in this case, a higher effect is related to the scanning speed rather than the layer thickness, as can be seen by comparing samples A with C (60–90 µm) and sample A with D (50–100 mm/s).

## Conclusions

In the present work, four scan tracks of 316L stainless steel were produced via L-PBF and modelled by numerical simulation to obtain a reliable method for the prediction of microstructural parameters, such as primary cellular arm spacing. The main conclusions can be summarized as follow:

- All samples resulted in cellular microstructure regardless of the process parameters used, which confirms the stability of this microstructure during the L-PBF processing of 316L stainless steel. In particular, for the process parameters under consideration, the PCAS was found to vary from 451 to 627 nm.
- From the analysis of temperature distribution within the melt pool, it emerged that melt pools of samples manufactured with the lower scanning speed resulted in depression zones persisting also after the laser scanning, due to the higher recoil pressure generated. Moreover, it is noted that scanning speed has greater effect than layer thickness on temperature distributions.
- The simulated results for PCAS showed high agreement with the experimental data, confirming the validity of the model and its potential in obtaining reliable results for microstructure prediction. The percentage difference between simulated and experimental PCAS values is in fact around 7%. Moreover, as a starting point for future studies, other mechanical properties in addition to microhardness should be evaluated from the simulated PCAS based on empirical formulas [2] and experimentally verified for specimens manufactured via L-PBF, in order to validate the numerical model also for yield and tensile strength prediction.
- The effect of layer thickness on cooling rate was investigated, revealing poor influence. The main effect on cooling rate was found to be the laser scanning speed. In fact, the cooling rate increased with increasing scanning speed. The maximum cooling rate was 8.15 × 10^6^ K/s, corresponding to a laser velocity of 100 cm/s and 90 μm layer thickness. However, further analysis on laser power effect should be considered in future studies.
- Experimental microhardness values reflect the general trend of PCAS. The prediction of microhardness based on cooling rate data following the model proposed by Ma et al. [2] slightly overestimates the microhardness of the samples, with a deviation in the range 1–11%. The prediction is less accurate for higher values of volumetric energy density, likely indicating that the equation could be optimized to extend the proposed microhardness model to volumetric energy density values above 78 J/mm^3^.
- In general, there was little effect on the hardness or PCAS from the two-layer thicknesses examined, pointing that the optimization of hardness or PCAS could be more readily achieved via scan speed and related control on microstructure.
